# Leveraging health infrastructure to optimize HPV vaccination for adolescents in Zambia: Protocol for an implementation study

**DOI:** 10.1371/journal.pone.0285031

**Published:** 2023-05-09

**Authors:** Sam Miti, Thembekile Shato, Comfort Asante, Ana Baumann, Gershom Chongwe, Patricia M. Bobo, Michelle I. Silver, Jean M. Hunleth

**Affiliations:** 1 Arthur Davison Children’s Hospital, Ndola, Zambia; 2 Tropical Diseases Research Center, Ndola, Zambia; 3 Division of Public Health Sciences, Department of Surgery, Washington University in St. Louis, St. Louis, Missouri, United States of America; 4 Implementation Science Center for Cancer Control and Prevention Research Center, Brown School, Washington University in St. Louis, St. Louis, Missouri, United States of America; 5 Ndola Teaching Hospital, Ndola, Zambia; 6 Ministry of Health Headquarters, Lusaka, Zambia; GERMANY

## Abstract

**Background:**

Cervical cancer is the leading cause of cancer death in Zambia, where HIV prevalence is also high (11.3%). HIV heightens the risk of developing and dying from cervical cancer. The human papillomavirus (HPV) vaccine can prevent 90% of cervical cancers, and in Zambia is recommended for adolescent girls ages 14–15 years, including those with HIV. Currently they mainly deliver HPV vaccination via school-based campaigns, which may exclude the most vulnerable adolescents—those out-of-school or who irregularly attend. Adolescents living with HIV (ALHIV) are more likely to have these vulnerabilities. Further, school-based campaigns are not tailored to the WHO-recommended HPV vaccination schedule for ALHIV (3 versus 2 doses). Integrating HPV vaccination into routine care in adolescent HIV clinics may ensure that ALHIV have access to vaccine at the WHO-recommended schedule. Such integration requires a multilevel approach, stakeholder engagement, and diversified implementation strategies, given known challenges of providing the HPV vaccine in LMICs, including Zambia.

**Methods:**

Our study aims to integrate HPV vaccination into routine care in adolescent HIV clinics. To achieve success, we will co-design a package of implementation strategies using a previously successful implementation research approach developed for cervical cancer prevention in LMICs: the Integrative Systems Praxis for Implementation Research (INSPIRE). INSPIRE is a novel, comprehensive approach to develop, implement, and evaluate implementation science efforts. Following key elements of INSPIRE, our specific aims are to: 1) Identify the unique multilevel contextual factors (barriers and facilitators) across HIV settings (rural, urban, peri-urban) that influence HPV vaccine uptake; 2) Use Implementation Mapping to translate stakeholder feedback and findings from Aim 1 into a package of implementation strategies to integrate HPV vaccine into HIV clinics; 3) Conduct a Hybrid Type 3 effectiveness-implementation trial to evaluate the package of multilevel implementation strategies for integrating HPV vaccine into HIV clinics.

**Discussion:**

Our research team has strong support, technical expertise, and resources (e.g., vaccines) from the Zambian Ministry of Health; and political will for scale-up. This stakeholder-based implementation model has the potential to be transported to HIV clinics across Zambia and serve as a model to address cancer prevention priorities for those with HIV in other LMICs.

**Trial registration:**

To be registered prior to Aim 3, when implementation strategies finalized.

## Background

Cervical cancer is the 4^th^ most common cancer among women globally and the leading cause of cancer death in Zambia, the site of this research [1–3]. The human papillomavirus (HPV) vaccine is a WHO-endorsed, evidence-based tool that can prevent 90% of cervical cancer cases by preventing HPV infection when administered prior to sexual activity and exposure to HPV. Thus, it is recommended for girls ages 9–14 [4, 5]. However, HPV vaccine coverage is low in low- and middle- income countries (LMICS) (16%) [6]. The WHO’s 2030 target is 90% of girls vaccinated fully by age 15 worldwide [7]. LMICs, including Zambia, are well below this target [6]. By 2019, only 31% of girls in Southern and Eastern Africa had received one dose; even fewer had two doses (20%), though coverage varies greatly by country [6]. Prevention is particularly critical for adolescents living with HIV (ALHIV) whose immune systems may be more susceptible to and less able to control HPV infections. HIV prevalence in Zambia is high, at 11.3% [8]. HIV prevalence among young women in Zambia (15–24 years) is 6%, more than double that of young men (2.8%), and an estimated 82,000 children ages 0–14 are living with HIV [3, 8]. HIV heightens the risk of developing and dying from cervical cancer, and 64% of cervical cancer cases in southern Africa occur in women who have HIV [9]. Given the long natural history of the cervical cancer development, preventing HPV infection needs to begin early.

At present, HPV vaccination in Zambia is mainly through school-based campaigns for 14–15 year old girls during Child Health Week, though efforts are also made to give vaccines at health facilities and in the community during that time. This campaign happens annually, so the 1^st^ and 2^nd^ doses of the vaccine are delivered 12 months apart. However, the WHO recommended regimen for ALHIV differs from regimens for adolescents who are HIV negative, both in dosage and in timing. In December 2022, the WHO updated their HPV vaccine recommendations to a 1–2 does schedule for girls ages 9–14, and emphasized the need to prioritize vaccinating those who are immunocompromised or living with HIV with a minimum of two doses, but recommends 3 doses when possible [10–13]. This creates challenges for school- and community-based campaigns. First, this would require disclosure of HIV or testing for HIV status, which would be inappropriate in school or community settings, causing unintended disclosure, stigma, and discrimination. Second, it would require a different vaccination schedule, which would be logistically challenging and again risk unintended disclosure of HIV status. Third, limited week-long school-based vaccination cannot reach the many adolescents who irregularly attend school [14–17]. Providing vaccination in pediatric and adolescent HIV clinics can close the gaps in vaccination. For ALHIV, HIV clinics can offer important sites for addressing these challenges (e.g., stigma, access, timing, health questions), reaching both in- and out-of-school ALHIV girls. In Zambia, because of the high prevalence of HIV, HIV clinics are integrated into all public hospitals and most clinics and therefore readily accessible. With multi-month drug dispensation, stable ALHIV attend HIV clinics every 6 months to collect ART and receive annual complete blood count, biochemistry, viral load and CD4 count monitoring, making HIV clinics a logistically feasible location to disperse HPV vaccinations. ALHIV often form long-term relationships with health providers through HIV care, and all clinics have HIV peer support groups for ALHIV. Such established, supportive infrastructure will provide the foundation for sustainably integrating HPV vaccination into clinics, providing a complement to school campaigns for this vulnerable population.

Our implementation study aligns with Zambia’s National Strategy for HPV Vaccination/Cervical Cancer Prevention and the 2019–2023 costing plan, which included an intention to roll out vaccination in health care centers [18]. Such restructuring has yet to be implemented, and our study can offer the Ministry of Health with data and testing needed to more rapidly scale up roll out in the country. Further, a review of studies carried out in countries in Lesotho, Cameroon, and Uganda identified that studies (n = 3) integrating HPV vaccination in clinics showed promising results (acceptability, coverage, vaccine series completion) [19–21]. Multilevel challenges exist at the systems level, such as inadequate infrastructure, insufficient human resources and staff capacity, high vaccine costs, and logistical barriers associated with delivery of a multi-dose vaccine [22–26]. Additionally, there are challenges at the individual and community-levels, including issues of access as well as limited knowledge, misperceptions, and stigma [23, 27, 28]. To address these challenges, we will co-design implementation strategies using a previously successful implementation research approach developed for cervical cancer prevention in LMICs: the Integrative Systems Praxis for Implementation Research (INSPIRE) [29]. INSPIRE is a novel, formal, and comprehensive framework to develop, implement, and evaluate implementation science efforts [29]. INSPIRE offers a four-phase approach that begins with defining the situation that needs addressing with stakeholders, and includes stakeholder-engaged research to make the system visible and co-design of implementation strategies. The final phase involves monitoring and evaluating the co-designed strategies. If successful, this stakeholder-based implementation model could be transported to HIV clinics across Zambia and serve as a model to address cancer prevention priorities for those with HIV in other LMICs.

We aim to co-design implementation strategies to integrate HPV vaccination into routine care in adolescent HIV clinics. Our proposal has strong support, technical expertise, and resources (e.g., vaccine) from the Zambian Ministry of Health, and political will for scale-up if successful. Following key elements of INSPIRE, our specific aims are to:

**Aim 1:** Identify the unique multilevel contextual factors (barriers and facilitators) across HIV settings (rural, urban, peri-urban) that influence HPV vaccine uptake.
**Aim 2:** Use Implementation Mapping to translate stakeholder feedback and findings from Aim 1 into selection of implementation strategies to integrate HPV vaccination into HIV clinics.
**Aim 3:** Conduct a hybrid type 3 effectiveness-implementation trial to evaluate the selected package of multilevel implementation strategies for integrating HPV vaccination into HIV clinics.

### Design and methods design overview

ALHIV are at high risk of developing HPV-related cancers. HPV-related cancers can progress more quickly and be more difficult to detect in people living with HIV. HPV vaccination is currently being implemented in many national plans, yet most current campaigns in LMICs are school-based, thus missing this most vulnerable population who may not attend school regularly and don’t have access to the necessary 3^rd^ dose. Vaccinating ALHIV in HIV clinics using the WHO-recommended 3-dose schedule will address this gap and is a needed step to address the inequities in cervical cancer burden among women living with HIV in Zambia. Engaging diverse stakeholders from the outset of the project (Aims 1 and 2) following the INSPIRE approach ensures that the package of implementation strategies selected for testing in Aim 3 are feasible, acceptable, and contextually appropriate.

### Setting and participating sites

In Zambia, HIV clinics are integrated into all public hospitals and most clinics and therefore readily accessible. With multi-month drug dispensation, stable ALHIV attend HIV clinics every 6 months to collect ART and receive annual complete blood count, biochemistry, viral load and CD4 count monitoring, making HIV clinics a logistically feasible location to disperse HPV vaccinations. ALHIV often form long-term relationships with health providers through HIV care, and all clinics have HIV peer support groups for ALHIV. Such established, supportive infrastructure will provide the foundation for sustainably integrating HPV vaccination into HIV clinics, providing an alternative to school campaigns for this vulnerable population.

We are partnering with adolescent HIV clinics in three distinct settings in and around Ndola, a city on the Copperbelt: 1) a large, urban tertiary referral hospital with the second largest pediatric and adolescent HIV care center in Zambia; 2) a peri-urban clinic that has youth-friendly services and is located in a low-income residential area; and 3) a hospital located in a rural, predominantly agricultural area. These sites were selected to represent the variation in HIV clinic sites and clinical delivery systems where ALHIV receive care across Zambia.

### Conceptual framework/approach

The Integrative Systems Praxis for Implementation Research (INSPIRE) is an implementation research approach for identifying and adapting a multilevel package of diverse implementation strategies in context through stakeholder engagement. INSPIRE was developed in and for LMICs, proposes a novel, formal, and comprehensive approach to develop, implement, and evaluate implementation science efforts specifically to address cervical cancer globally [29]. To develop context-adapted implementation strategies that are both scalable and sustainable, INSPIRE uses stakeholder engagement and systems thinking, focusing on settings and systems using the Consolidated Framework for Implementation Research (CFIR) [30, 31] and comprehensively assessing outcomes by focusing on reach, effectiveness, adoption, implementation, and maintenance (RE-AIM) [29, 32]. INSPIRE was developed and used with great success in the Peruvian Amazon to improve cervical cancer screening, where its use more than doubled screening among 30–49 year old women by implementing HPV-based testing, while also reducing the time to receiving results, and almost doubling treatment completion (personal communication, Dr. Patti Gravitt). We propose to use this successful approach to improve cervical cancer vaccination among ALHIV in Zambia.

The INSPIRE approach is comprised of an initial ‘Hub’ step followed by a 4-phase process [29]. See Table 2 for description of the Hub and 4-phase process and how we map our aims and activities onto the approach. Notably, we have completed the critical ‘hub’ activities (e.g., defining situation with stakeholders) of the INSPIRE approach (Table 1). The impetus for initiating hub activities was twofold. First, Dr. Miti and his colleagues observed substantial adolescent and community vaccine hesitancy during their HPV vaccination campaigns in schools in Copperbelt Province, Zambia. Community members expressed concern about HPV vaccination causing infertility and cancer. Adolescents showed their hesitancy by skipping school or running away from vaccinators (in one school, they climbed through windows). Second, Dr. Miti and his clinical partners have observed that many of their patients are missing the HPV vaccination, and that their HIV patients are missing out on the 3-dose schedule they require. Given these factors, Dr. Miti, in coordination with Drs. Hunleth and Silver, met with Ministry of Health (MoH) officials to identify their program priorities to address this hesitancy and increase vaccination access and uptake. Together, the MPIs and the MoH identified a need for implementation strategies to roll-out HPV vaccines into health care facilities.

**Table 1:** Study aims and activities mapped onto INSPIRE [39].

	Definition	Aim and Study Activities
Hub	Define situation with stakeholders; launch project	Completed- MPIs worked with MoH to identify need for implementation strategies to roll-out of HPV vaccine into health care centers and develop this proposal
Phase I	Develop mental models of system; establish narrative and stakeholder perceptions; make system visible	Aim 1- Use Implementation Mapping tasks 2 for Rapid Ethnographic Assessment to collect stakeholder perceptions and clinic observations to understand the system
Phase II	Engage stakeholders in group model building; share, test, revise system/process maps; define and localize system behaviors contributing to problem; find leverage for change	Aim 2- Use Implementation Mapping tasks 2 and 3 to identify implementation outcomes, performance objectives & determinants, create matrices of change to find leverage
Phase III	Stakeholder-designed implementation plan; infrastructure modifications, training dissemination plan development; implement changes	Aim 2- Use Implementation Mapping tasks 3 and 4 for stakeholders to select implementation strategies and for team to develop protocols and study materials; Aim 3- Hybrid type 3 trial to implement changes
Phase IV	Ongoing M&E using stakeholder defined implementation outcome metrics; share M&E with stakeholder; reinitiate INSPIRE cycle if indicated for new or unresolved problem	Aim 3- RE-AIM for outcome evaluation; dissemination of findings

In Aim 1, we will carry out Phase I of INSPIRE (i.e., Understand the System) [29] using ethnographic methods (e.g., key informant interviews, group discussions, and observations) to deepen existing understandings of barriers and facilitators while cultivating multi-level stakeholder buy-in (e.g., policymakers, clinical staff, community leaders, adolescents, and household members of adolescents). Table 2 describes in more detail these stakeholders and their roles in this study. We will use established rapid ethnographic assessment (REA) tools as outlined by Sangaramoorthy and Kroeger [33] and others [34] that are commonly used in intervention research in Zambia [35] and have been used extensively by Dr. Hunleth in clinical, community, household, urban, peri-urban, and rural settings in Zambia [36–45] REA is needed to facilitate a holistic picture of contextual and setting influences that account for different stakeholder roles, responsibilities, priorities, and perspectives. We will use REA, focusing on the three domains from the Consolidated Frameworks for Implementation Research (CFIR) [30] emphasized in INSPIRE Phase 1: outer setting, inner setting, and characteristics of individuals. Using these CFIR domains will enable comparison among our 3 diverse study sites: urban, peri-urban, and rural. We will analyze the REA at each study site separately, and then compare and contrast REA findings across sites. Such data and analysis will identify barriers, facilitators, and priorities that cross-cut setting and those that are setting specific. This information will be critical to designing a package of strategies to implement across sites that is both standardized and flexible.

**Table 2:** Stakeholder constituents, roles, and modes of participation.

Stakeholder Category	Description/Constituents	Role	Mode of Participation
Governmental and Non-Governmental Organizations	Individuals and entities involved in adolescent welfare and programming, particularly for ALHIV, at the National, Provincial, and/or District level	Provide knowledge of institutional processes, challenges, and capacities Provide insight into logistic features of the health systems to assess potential for various infrastructure modifications Those constituting Research Advisory Boards will also: Assist in the creation of monitoring mechanisms and metrics used in ongoing evaluation of intervention Provide technical assistance, training, and oversight during integration Prepare various parts of the systems for integration Sustain the intervention throughout changes in government administration Assist in making research processes, objectives, and goals understood and accessible for community members	Stakeholder Engagement Meetings, Kick-off Meetings, Research Advisory Boards, Dissemination Meetings AIM 1, AIM 2, AIM 3
Clinical*	Clinical staff who work in a range of positions integral to clinical site operations and who regularly engage adolescents, particularly ALHIV, in their work	Assist in auditing current health system behavior, structures, and flows within each clinic Provide feedback to identify restructuring opportunities for better integration of HPV vaccination into the clinical space Modify/adapt clinical infrastructure Assist in the identification and location of system barriers and identify “leverage for change” Ensure the strategies are logistically feasible to deliver and sustain on a clinical level Assist in the recruitment of intervention participants (ALHIV) Assist in the implementation and administration of HPV vaccination in clinical space	Focus Group Discussions, Key Informant Interviews, Community Advisory Boards AIM 1, AIM 2, AIM 3
Adolescent girls living with HIV*	Girls ages 9–14 who are living with HIV and receiving care at one of the site clinics	Share their experiences navigating the health system Share knowledge and experiences related to HPV vaccination among ALHIV Identify individual and local health priorities and challenges (particularly regarding HPV vaccination) Ensure strategies for delivery of HPV vaccination are acceptable Provide feedback on the implementation of strategies	Focus Group Discussions, Key Informant Interviews AIM 1, AIM 2, AIM 3
Household members*	Kin, guardians, parents, or HIV-negative adolescents living with an ALHIV	Offer critical insight into household discourse and perspectives on HPV vaccination Share their experiences navigating the health system, particularly for adolescent health care Share perspectives on facilitators and barriers to HPV vaccination Share perspectives on strategies for delivery of HPV vaccination	Focus Group Discussions, Key Informant Interviews AIM 1, AIM 2, AIM 3
Community*	People known to hold knowledge/influence in the study sites, such as elders, traditional healers, teachers, adolescents, etc.	Identify community-level facilitators, barriers, and preferences, particularly related to adolescent health and HPV vaccination Identify issues relevant to their communities and the study sites that affect adaptation Share perspectives on strategies for delivery of HPV vaccination	Focus Group Discussions, Key Informant Interviews AIM 1, AIM 2

Aim 2 will address Phases II and III of INSPIRE (i.e., Find Leverage and Act Strategically) through the use of implementation mapping to engage stakeholders in workshops to select and adapt evidence-based implementation strategies to target the multilevel contextual factors influencing HPV vaccination as identified in Aim 1. Implementation mapping is a step of intervention mapping focused on using theory to systematically select implementation strategies that can target multiple levels of influence to increase the adoption, implementation, and sustainability of an existing evidence-based intervention (in this case, HPV vaccination) [46, 47]. Like the INSPIRE approach that informs our overall study, implementation mapping also emphasizes principles of participatory action research (PAR). It involves 5 tasks: 1) Conduct a needs and assets assessment, identify barriers and facilitators and program adopters and implementers; 2) State implementation outcomes and performance objectives determinants, and create matrices of change objectives; 3) Choose mechanisms of change and select or create implementation strategies; 4) Produce implementation protocols and materials; and 5) Evaluate implementation outcomes [46, 47]. Implementation mapping is an iterative process, allowing for continued integration of feedback from stakeholders and refinement or adaptions to strategies as needed.

In Aim 3, we will carry out INSPIRE Phases III and IV (i.e., Act Strategically, and Learn and Adapt) [29]. by implementing the multilevel intervention package co-created with our multilevel stakeholders in Aim 2 and then evaluating implementation, HIV, and HPV outcomes. We will conduct a Hybrid Type 3 trial (focused on implementation outcomes while also collecting effectiveness outcomes) to deliver HPV vaccination in 3 adolescent HIV clinics (rural, urban, peri-urban). Evaluation of implementation, HPV vaccination, and HIV outcomes (viral load, CD4, ART adherence) will be guided by the RE-AIM approach [48]. The specific package of multilevel implementation strategies will be determined in Aims 1 and 2, but below we outline our planned intervention and procedures. The primary setting of the intervention will be the three HIV clinics selected. The intervention package will be standardized and flexible. There will be many similarities in the intervention package across the sites. However, as the intervention package is being co-created with stakeholders separately at each site, we anticipate distinct differences due to the differences in context. The differences may be in which strategies are selected or the way they are implemented. Nonetheless, we expect all sites to have strategies focused on individuals/patients, households, providers, clinics, and the community level.

All study activities used in this study will be carried out in accordance with ethical guidelines and regulations in the United States and Zambia. This study has been approved by the Research Ethics Committee at the Tropical Diseases Research Centre (Ref: TDREC/053/11/22), the Zambian National Health Research Authority (NHRA000013/19/01/2023), and Washington University’s Institutional Review Board (IRB ID #: 202211001). We will obtain oral informed consent or assent (minor participants) from all study participants. For participants under 18 years of age, we will obtain oral informed consent from their legal guardians.

### Aim 1: Identify the unique multilevel contextual factors (barriers and facilitators) across HIV settings (rural, urban, peri-urban) that influence HPV vaccine uptake

#### Participants

Within each of the three sites, we will engage four different types of stakeholders as participants to ensure that we attain diverse, multilevel perspectives and buy-in: clinical, ALHIV, household, and community (refer to Table 1 for more details). Clinical stakeholders include key staff working with ALHIV and we will purposefully interview clinical stakeholders with different roles. ALHIV stakeholders will consist of girls (ages 9–14). While we acknowledge that boy ALHIV hold important perspectives, we focus in this study on girl ALHIV because, at present, the Zambian Ministry of Health only plans to deliver vaccination to girls (due to supplies). Household stakeholders are those people living with an ALHIV girl (9–14). Household stakeholders offer critical insight as they may shape household perspectives on HPV vaccination or help ALHIV access care. Household stakeholders include- along with kin, guardians, or parents- other adolescents beyond ALHIV who may need HPV vaccinations themselves. Community stakeholders include people known to hold power in Zambian communities, such as elders and traditional healers, as well as teachers and other professionals. Eligibility for all stakeholders is limited to residing or working in these three pre-determined clinical site settings. Further eligibility for clinical stakeholder will include working in or adjacent to services offered to ALHIV. For ALHIV, eligibility will include being: 1) in treatment at the site’s HIV clinic, 2) between ages of 9 and 14, and 3) female. Eligibility as household stakeholder will be assessed via self-report and includes: 1) living in one of the 3 HIV clinic catchment areas and 2) living with a girl ALHIV between ages of 9 and 14.

#### Procedure

To ensure that the REA produces usable implementation data for Aims 2 and 3 and allows us to reach saturation and compare across sites, we focus the REA using the CFIR domains (outer setting, inner setting, intervention characteristics, characteristics of individuals) [32] that Gravitt et al. identified as critical to Phase 1 research in INSPIRE. See Table 3 for outlined toolkit, which includes key informant interviews, group discussions, and observations. Key informant interviews are aimed at eliciting everyday experiences and needs. We will follow a semi-structured REA interview guide. Guides have four types of questions (descriptive, experience, perception, structured) that allow conversation to flow yet remain focused. Group discussions facilitate interactions among people that aim to elicit norms and values and also generate debate about an intervention [33]. PI Hunleth has identified group discussions, carried out in de-personalized ways sensitive to group dynamics, as critical to identifying norms about health interventions on sensitive and power-laden topics, such as provider-patient relations and adolescent health concerns. As we have done in past studies, we will use a combination of discussion (e.g., Can you describe what you have heard about the HPV vaccination?) and participatory activity, such as role-play (e.g., for ALHIV: Act out coming to the clinic for HIV treatment) [39]. Observations provide a critical form of evidence about the inner and outer settings, especially issues of structural characteristics and compatibility that may not be easily gleaned from interviews or group discussions. Trained observers will carry out observations in key clinical sites and during clinic meetings and gatherings. Observation protocols will be standardized across sites to facilitate comparison, but they will also be flexible to consider settings and gatherings that are site specific. Observations will be documented using structured fieldnote guides and analyzed immediately following Vindrola-Padros and the Rapid Research Evaluation Lab’s framework for synthesizing findings and identifying key topics [34].

**Table 3:** Rapid ethnographic assessment toolkit by stakeholder and site.

	CLINICAL STAKEHOLDERS	ALHIV STAKEHOLDERS	HOUSEHOLD STAKEHOLDERS	COMMUNITY STAKEHOLDERS	CLINICAL SITE SETTING
ACTIVITIES	Key informant interviews Characteristics of individuals Inner setting Outer setting Cultivate buy-in	Group discussions Outer setting Inner setting	Group discussions Outer setting Inner setting	Group discussions Outer setting Cultivate buy-in	Observation types: Immersion, planned, instant record Inner setting Characteristics of individuals Cultivate buy-in
SAMPLING BY SITE	10 interviews Nurses Doctors Community health workers Pharmacists	2 groups (n = 16–20 ALHIV girls) Girls 9–11 yrs Girls 12–14 yrs	3 groups (n = 24–30) Men Women Girls 9–14 yrs (HIV-)	2 groups (n = 16–20 people) Men Women	2 week immersion during interviews and group discussions 5 planned meetings or events attended 10 instant records of HIV clinic

#### Analysis

REA analysis occurs throughout data collection using structured analysis sheets and framework analysis [34]. Analysis sheets will be structured using CFIR domains (outer setting, inner setting, intervention characteristics, and characteristics of individuals) and sub-domains (e.g., patient needs and resources in outer setting) [32], but will remain flexible to account for emerging themes. Analysis sheets will be developed specific for each data collection tool and stakeholder group and we will compile the data using NVivo20’s framework matrix capabilities. We will compare findings across stakeholder groups and data collection tools for each site, as well as compared between sites, allowing for an understanding of barriers and facilitators that cut across sites and those that are site and setting specific. Community advisory boards will assist with interpretation of findings.

#### Advisory board

During Aim 1, we will also convene advisory boards. Advisory board members will not participate as research participants. However, they are critical to REA research, as members can advise on toolkit appropriateness, recruitment, and interpretation. We will convene two different types of advisory boards during the REA that will continue to provide advisory roles throughout Aims 2 and 3: a research advisory board at the provincial level and community advisory boards in each clinical setting site. The research advisory board will be comprised of Ministry of Health and Ministry of Education employees and clinicians in technical support roles, and will advise on systems- and policy-level issues. The community advisory boards will be comprised of clinicians, ALHIV, and household and community members in each clinical site setting.

### Aim 2: Use Implementation Mapping to translate stakeholder feedback and findings from Aim 1 into selection of implementation strategies to integrate HPV vaccination into HIV clinics

#### Participants

During this aim, we will facilitate workshops to engage with stakeholders from Aim 1 (ALHIV, clinical, household, community) to identify leverage for change, and co-create the implementation plans (INSPIRE Phases II and III). We will then present the package of selected implementation strategies to our community advisory boards (CABs) and research advisory board (RAB) for feedback.

#### Implementation mapping tasks

Implementation mapping consists of 5 tasks (steps) to engage stakeholders in workshops to select and adapt evidence-based implementation strategies to target the multilevel contextual factors influencing the evidence-based intervention. Table 4 provides a summary of the activities planned to complete each of the 5 implementation mapping tasks.

**Table 4:** Implementation mapping tasks and study activities for each task.

Task	Description	Study Activities to Complete Task
1	Conduct needs and assets assessment	Rapid Ethnographic Assessment with stakeholders (Aim 1)
2	Identify adoption and implementation outcomes, performance objectives, and determinants; create matrices of change	MPIs and study team meet to synthesize Aim 1 findings and identify implementation outcomes, performance objectives & determinants, create matrices of change
3	Choose theoretical methods; Select or create implementation strategies	Study team generates list of potential implementation strategies; Workshop for stakeholders to select and adapt implementation strategies
4	Produce implementation protocols and materials	MPIs and study team create protocols and materials, present to stakeholders, RAB, and CABs and iterate based on feedback
5	Evaluate Implementation Outcomes	RE-AIM for outcome evaluation (Aim 3)

*Task 1*: *Needs and assets assessment*. We will use thematic analysis to summarize the barriers and facilitators identified in Aim 1 through our REA and from our stakeholders. We will identify which barriers and facilitators are common across settings and which are unique to each site. We will also classify the level(s) (individual, clinical, community) at which each barrier and facilitator operates and influences.

*Task 2*: *Identify adoption and implementation outcomes*, *performance objectives*, *and determinants; create matrices of change*. The MPIs Hunleth, Miti, and Silver will meet with the study team to identify the *performance objectives* (specific tasks and actions) that are required at each level (individual, clinical, community) to implement HPV vaccination and achieve our intended *implementation outcome* of successfully integrating HPV vaccination into adolescent HIV clinics to prevent cervical cancer in ALHIV. We will use this information to create “matrices of change” for each level that show what factors needs to be changed in order for each performance objective to be met. These matrices and needed changes will guide the selection of implementation strategies in conjunction with our stakeholders in Task 3.

*Task 3*: *Choose theoretical methods; select or create implementation strategies*. The study team will generate a list of potential implementation strategies based on the matrices of change created in Task 2, pulling from the Expert Recommendation for Implementing Change (ERIC) taxonomy [49]. and from previously successfully evidence-based strategies for vaccination in LMICs. We will then facilitate interactive half-day workshops with stakeholder groups at each site for so they can prioritize the identified barriers and facilitators and guide selection and adaptation of implementation strategies best suited to meet their needs and the identified challenges. Multiple evidence-based implementation strategies will be selected to target each performance objective and associated changes identified for each level in the previous task; many of these strategies will influence multiple objectives simultaneously. We will ensure that the final implementation package includes a range of implementation strategies that address the CFIR constructs of the outer setting (particularly ALHIV needs), as well as the inner setting, especially readiness for implementation (leadership engagement, available resources, and access to knowledge and information), and the intervention characteristics (such as knowledge and acceptance of the vaccine). The final list of strategies chosen by stakeholders in these workshops will guide the development of study protocols and materials for the intervention in Task 4.

*Task 4*: *Produce implementation protocols and materials*. Based on the stakeholder selected strategies from Task 3, the study team will define selected strategies, develop study protocols, and prepare all necessary study materials. We will then review and refine all materials with our stakeholders, before presenting to our CABs and RAB for additional feedback, and refinement as needed. The final results of this task will form the basis of the multilevel Hybrid Type 3 trial conducted in Aim 3.

*Task 5*: *Evaluate implementation outcomes*. Evaluation of implementation outcomes, as selected and refined with stakeholders in earlier tasks, will occur in Aim 3, using the RE-AIM framework (Table 5), after implementation of our multilevel intervention to integrate HPV vaccination into adolescent HIV clinics.

**Table 5:** RE-AIM outcome evaluation components [32, 51].

RE-AIM Dimensions	Quantitative Evaluation Metric(s) [data source]	Qualitative Evaluation Questions [data source]
**Reach**	# and % of ALHIV (and/or eligible household members) offered vaccine Same as above for initiating vaccine series Characteristics of those who get at least 1 dose and those who do not [Chart review data]	What factors contribute to participation of [ALHIV or eligible household members]? What might have been done to reach more [ALHIV or eligible household members]? [key informant interviews]
**Effectiveness**	# and % of ALHIV (and/or eligible household members) who complete vaccine series HIV outcomes (viral load, CD4) [Chart review data] ART adherence [survey]	Did the intervention work to effect the outcomes identified? What unanticipated factors contributed to the outcomes? Are the outcomes meaningful to [clinicians, patients]? What were the unintended consequences of the strategies used? [observation; key informant interviews]
**Adoption**	# and % of providers offering vaccine [Clinic staff and administration surveys]	What factors contributed to the clinic and staff taking up the strategies? What barriers and facilitators interacted with adoption? Was adoption partial or complete? [observation; key informant interviews]
**Implementation**	Proportion of completed vaccine series out of total initiated Timing between doses [Chart review data]	How was the intervention implemented? By whom and when? What influenced implementation or lack of implementation? How and why were adaptations made over time? [observation; key informant interviews]
**Maintenance**	Number of doses administered per month for the 12-months after active intervention	Is the intervention package sustained? Which strategies are sustained, discontinued, or modified? [observations; key informant interviews]

#### Logic model creation

**Fig 1:**
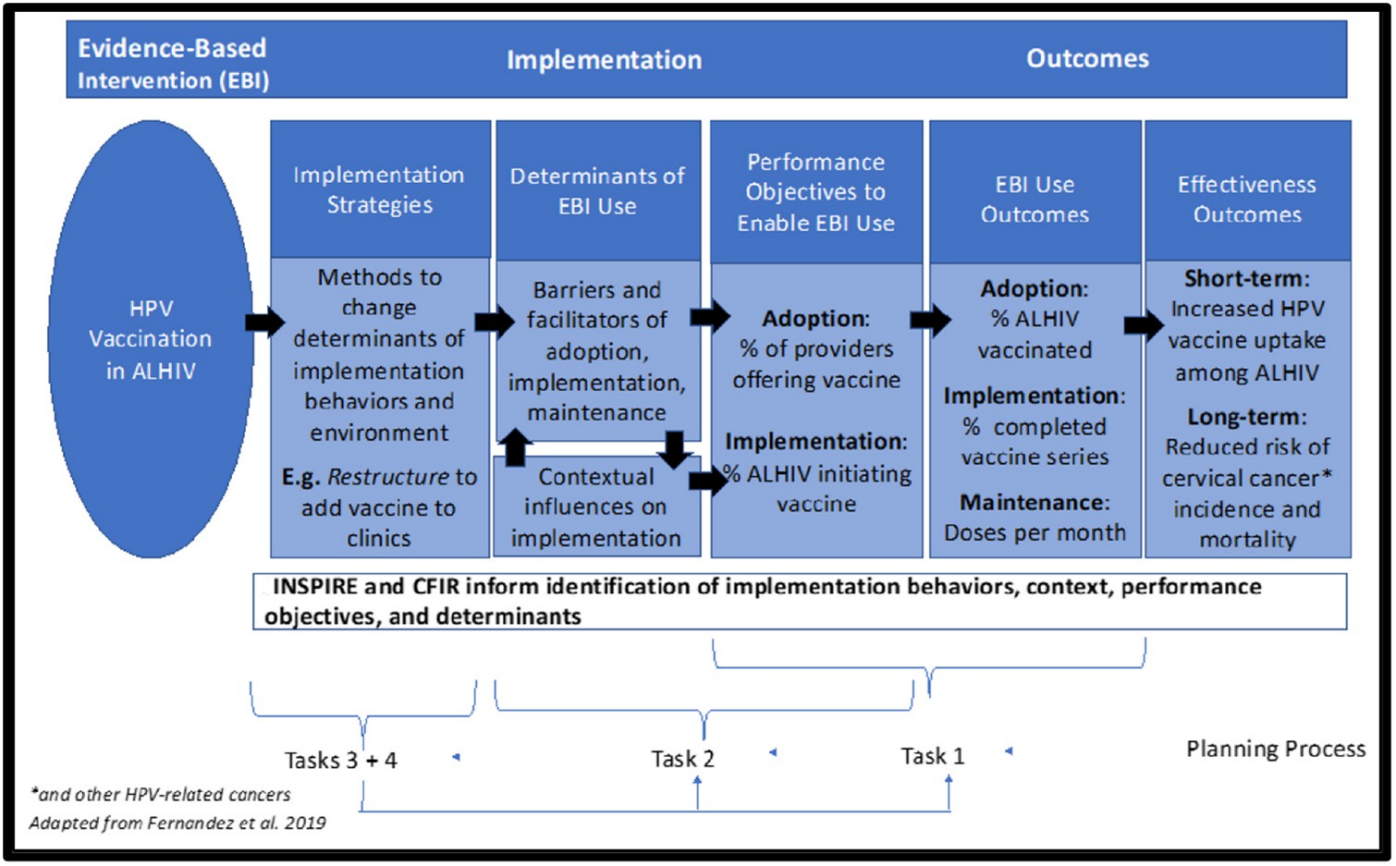
Logic model for implementation mapping.

Using the outputs of each task in our implementation mapping process we will create a logic model to visually depict the process of implementing our EBI (HPV vaccination) to achieve our desired outcomes. Fig 1 illustrates an example of the logic model we will create in the implementation mapping process, using examples of the potential types of performance objectives and EBI Use Outcomes we will measure. Our final logic model will include all identified strategies, determinants, objectives and outcomes. Each task in our implementation mapping process will be informed by INSPIRE and CFIR to guide identification of strategies that aid in implementing vaccination, context, performance.

#### Expected outcomes and markers of success

Upon completion of this aim, we will have a fully developed study design (including protocols and study materials) for a Type 3 Hybrid Trial of a multilevel package of implementation strategies to integrate a 3-dose HPV vaccine series into 3 adolescent HIV clinics in and around Ndola, co-created with our Zambian partners and stakeholders. This aim will be considered successful if there is participation across all stakeholder groups (ALHIV, clinical, household, community), resulting in a feasible and mutually agreed upon package of implementation strategies for integrating HPV vaccination into existing HIV infrastructure that is ready for testing in Aim 3. By developing a stakeholder informed implementation strategy, we maximize the possibility of successful implementation and sustainment.

### Aim 3: Conduct a hybrid type 3 effectiveness-implementation trial to evaluate the selected package of multilevel implementation strategies for integrating HPV vaccination into HIV clinics

#### Participants

The participants in this aim will be clinical staff and other administrators, as well as girls who receive the vaccination as part of the intervention. Eligibility for vaccination include, being a girl between the ages of 9 and14 years who is living with HIV and attending any of our 3 clinic sites. Any additional 9–14 year old female household members will also be eligible for HPV vaccination regardless of HIV status.

#### Procedure

Exact study protocols and procedures for introducing HPV vaccination into adolescent HIV clinics will be developed during implementation mapping in Aim 2 based on the implementation strategies selected by stakeholders in order to tailor protocols to site and context. The HPV vaccination intervention itself will involve inviting all eligible clinic patients to receive HPV vaccination and to further recruit HIV negative or positive age-eligible household members. HIV-positive participants will be offered a 3-dose regimen and HIV negative participants a 2-dose regimen, per WHO recommendations. Staff in each clinic involved in delivery of the vaccine will be trained about the vaccine, its importance, how to introduce it to patients, and how to store and administer the vaccine. Additional trainings and implementation strategies (as selected in Aim 2) will also be introduced at the appropriate times. We anticipate including additional intervention components aimed at recruiting eligible household members for vaccination as well.

Since this is a Hybrid Type 3, our primary interest is in implementation outcomes, with a secondary focus on effectiveness outcomes [50]. To evaluate outcomes using RE-AIM, we will use quantitative and qualitative methods, including chart review, surveys, observations, and interviews at the clinic, provider, and patient levels [32, 48]. Per INSPIRE, exact protocols, even for recruitment, are determined collaboratively (Aim 2 of this proposal). However, we expect to have embedded/local research staff work with clinics to recruit and consent girls, and recruit household members, for the study during their routine appointments at the HIV clinic. If girls choose to get vaccinated, they will be asked to complete brief exit surveys after each dose received. A sample of girls will be chosen for key informant interviews. Clinic staff and other clinical administrators will also be invited to complete brief surveys and interviews at three points during implementation—initial implementation, mid-point, and post-implementation—unless our observations identify other critical time points at the site(s)—to identify any bottlenecks or other challenges that need to be addressed. We will also carry out observations using a structured guide to identify adaptations made during the intervention at each site and examine questions related to reach, effectiveness, adoption, implementation, and maintenance. Chart review will be used to obtain patient demographics and HIV outcomes (adherence to ARTs, viral load, CD4 count), and for those who do get vaccinated, HPV vaccination data (uptake, number of doses, dose intervals) as well. For more details, see Table 5.

#### Quantitative data and analysis

The Zambian National Plan only includes HPV vaccination in schools and not in any clinics in the country. Therefore, the baseline status is no vaccination in any of these clinics, so we will be able to assess uptake and reach based on the amount of vaccination given in the clinics during our study. Our post-vaccination surveys will primarily include multiple choice responses and Likert scales, and will include questions about their experience with the intervention and vaccination and ask about ART adherence, barriers to vaccination, and care for girls living with HIV. Surveys will use validated measures where possible and will be pre-tested for comprehension and acceptability. For feasibility, we will intentionally keep surveys short. Our initial analyses will be descriptive, summarizing participant characteristics (including HIV-related variables) and attitude towards HPV vaccination overall and experience receiving the vaccine at the HIV clinic. We will then perform quantitative analyses using t-tests for continuous variables and chi-square tests for categorical variables to examine associations between patient characteristics, number of doses, and survey responses, followed by regression analyses. For all quantitative analyses, we will set a significance threshold of p<0.05. With a sample size of at least 600 surveys from vaccines overall, we can ensure that the margin of error will be no more than +/-4% at a 95% confidence level, so with at least 200 respondents per site, we will have a margin of error that is no more than +/-7% at a 95% confidence level. Additional quantitative analyses will include summarizing survey results from clinic staff and administrators as well as comparing HIV outcomes between girls who do and do not get the HPV vaccine, accounting for doses.

#### Qualitative data and analysis

Qualitative data will include observations and key informant interviews with providers and the girls receiving the intervention (both ALHIV and eligible household members). Qualitative data are critical to understanding implementation outcomes as they will allow us to understand how and why the intervention packages worked across RE-AIM dimensions and to examine the relations between the dimensions and strategies [32, 51]. In line with INSPIRE, the qualitative data collection guides will be focused using two CFIR domains: characteristics of the intervention and process of implementation (see the qualitative evaluation questions in Table 5). As we did in Aim 1, we will use observation protocols that are standardized across sites to facilitate comparison, but that will also be flexible to consider the differences in the packages of strategies that are site specific. Observations will be documented using structured fieldnote guides and analyzed immediately following Vindrola-Padros and the Rapid Research Evaluation Lab’s framework for synthesizing findings and identifying key topics [34]. Observational data collection will continue throughout the implementation timeframe to examine changes through time. We will carry out short (<30 min), structured, key informant interviews at three points during implementation—initial implementation, mid-point, and post-implementation—unless our observations identify other critical time points at the site(s). We anticipate a total of 12 interviews (6 provider; 6 patient) per time period per site will achieve saturation. Qualitative data analysis will occur similarly to Aim 1, with data analyzed as it is collected. We will use a framework matrix for each site and compare findings among sites.

While quantitative and qualitative data will be analyzed separately, we plan to merge the results to expand and/or confirm understandings of implementation [52].

## Considerations and limitations

The investigators have a strong track record of research in Zambia and an established working relationship with the Ministry of Health and research sites. Carrying out such a contextualized approach to implementation is critical to address issues of appropriateness, acceptability, feasibility, sustainability, and scalability, but it is necessarily messy, and we have anticipated a number of alternative approaches in our design. First, we anticipate that our recruitment plan is feasible based on our permissions from the Ministry of Health and prior experience. However, should challenges arise at the identified sites, we have permission from the Permanent Secretary to work with other clinics and hospitals in the province. Second, patients may decline the intervention. To mitigate against this, we included substantial ALHIV and other stakeholder engagement. Third, the intervention may exceed clinic capacity. Again, we have included substantial stakeholder engagement to design feasible implementation packages.

## Discussion

This project proposes an impactful and scalable approach to addressing cervical cancer risk among people with HIV in Zambia and LMICs more broadly. Cervical and other HPV-related cancers disproportionately impact people living with HIV. HPV vaccination is a key pillar of the World Health Organization’s strategy to eliminate cervical cancer, but implementation is lagging, particularly for especially vulnerable populations. Implementation studies of HPV vaccination have not leveraged HIV clinic structures in countries with high HIV burdens, instead focusing on schools. While schools can reach a broad population, school-based vaccination alone excludes those most at risk of suffering and dying from cervical cancer, like ALHIV, and does not follow the WHO 3-dose schedule for ALHIV. Our novel leveraging of adolescent HIV care and treatment infrastructure—using an implementation approach with substantial stakeholder input, buy-in, and co-creation—will push the field forward into robust, sustainable, stigma-reducing, and contextually-informed interventions to narrow inequities in vaccine coverage and ultimately reduce cervical cancer. In addition, the clinic setting will allow ALHIV to receive the WHO recommended 3^rd^ dose of the HPV vaccine without risk of stigma or status disclosure at school.

We utilize INSPIRE to leverage the unique resources and infrastructure in Zambia to co-create a diversified package of implementation strategies for integrating HPV vaccination into HIV clinics. Our innovative application of INSPIRE—designed and successfully used as a framework for implementing changes to cervical cancer screening in Peru—to integrating HPV vaccination into HIV care clinics is readily scalable to HIV clinics across Zambia and LMICs more broadly, as our approach integrates local context by design from beginning to end. In particular, our multilevel approach includes ALHIV, household and community members, and healthcare providers, as well as policymakers and other power holders. We recognize the range of factors that might influence an HPV vaccination program in HIV clinics. Our inclusion of ALHIV and a range of stakeholders, including policymakers, is both significant and innovative. The rapid ethnographic, implementation mapping, and multilevel approach, while each increasingly common on their own, have been underutilized in combination as complementary approaches in implementation research to date.

## Supporting information

S1 File (DOCX)

## Abbreviations

ALHIV: adolescents living with HIV
ART: antiretroviral therapy
CFIR: Consolidated Framework for Implementation Research
HPV: human papillomavirus
INSPIRE: The Integrative Systems Praxis for Implementation Research
REA: rapid ethnographic assessment
RE-AIM: reach, effectiveness, adoption, implementation, and maintenance
